# The Prevalence of Mycoplasma Pneumoniae Among Children in Beijing Before and During the COVID-19 Pandemic

**DOI:** 10.3389/fcimb.2022.854505

**Published:** 2022-04-29

**Authors:** Yuan Cheng, Yijing Cheng, Shuzhi Dai, Dongqing Hou, Menglei Ge, Yi Zhang, Lei Fan, Yingying Pei, Li Yu, Guanhua Xue, Lijuan Ma, Hongmei Sun

**Affiliations:** ^1^ Department of Clinical Laboratory, Children’s Hospital Affiliated to Capital Institute of Pediatrics, Beijing, China; ^2^ Child Health Big Data Research Center, Capital Institute of Pediatrics, Beijing, China; ^3^ Department of Bacteriology, Capital Institute of Pediatrics, Beijing, China

**Keywords:** children, COVID-19 pandemic, restrictive measures, *Mycoplasma pneumoniae*, epidemic

## Abstract

**Objective:**

*Mycoplasma pneumoniae* (*M. pneumoniae*) is an important pathogen of community acquired pneumonia. With the outbreak of coronavirus disease 2019 (COVID-19), the prevalence of some infectious respiratory diseases has varied. Epidemiological features of *M. pneumoniae* in children from Beijing (China) before and during the COVID-19 pandemic were investigated.

**Methods:**

Between June 2016 and May 2021, a total of 569,887 children with respiratory infections from Children’s Hospital Affiliated to Capital Institute of Pediatrics (Beijing, China) were included in this study. *M. pneumoniae* specific-IgM antibody in serum specimens of these patients was tested by a rapid immunochromatographic assay kit. The relevant clinical data of *M. pneumoniae*-positive cases were also collected, and analyzed by RStudio software.

**Results:**

The results showed that 13.08% of collected samples were positive for *M. pneumoniae* specific-IgM antibody. The highest annual positive rate was 17.59% in 2019, followed by 12.48% in 2018, 12.31% in 2017, and 11.73% in 2016, while the rate dropped to 8.9% in 2020 and 4.95% in 2021, with significant difference. Among the six years, the positive rates in summer and winter seasons were significantly higher than those in spring and autumn seasons (*p* < 0.001). The positive rate was the highest in school-age children (22.20%), and lowest in the infant group (8.76%, *p* < 0.001). The positive rate in boys (11.69%) was lower than that in girls (14.80%, *p* < 0.001). There were no significant differences in different seasons, age groups, or genders before and during the COVID-19 pandemic (*p* > 0.05).

**Conclusions:**

Our study demonstrated that an *M. pneumoniae* outbreak started from the summer of 2019 in Beijing. After the COVID-19 pandemic outbreak in the end of 2019, the *M. pneumoniae* positive rates dropped dramatically. This may be due to the restrictive measures of the COVID-19 pandemic, which effectively controlled the transmission of *M. pneumoniae*. The relationships between *M. pneumoniae* positive rates and season, age, and gender were not statistically significant before and during the COVID-19 pandemic.

## Introduction

*Mycoplasma pneumoniae* (*M. pneumoniae*) is an important pathogen of community acquired pneumonia (CAP), especially in children. Up to 40% of CAP in children, especially in school-age children, are caused by *M. pneumoniae* (Brown et al., 2016). This pathogen can be transmitted by droplets through the respiratory tract, easily causing respiratory tract infection (Waites et al., 2017). The *M. pneumoniae* epidemic may occur at an interval of 3-7 years and may last for up to 2 years (Lenglet et al., 2012; Yan et al., 2016). In addition, its epidemiologic characteristics may vary in age, gender, geographic area, etc., and may have a seasonal trend as well (Yan et al., 2019; Zhang L. et al., 2021). Previous studies showed that there was an *M. pneumoniae* epidemic from 2015 to 2016 (Brown et al., 2016; Yamazaki and Kenri, 2016; Yan et al., 2016). It was estimated that 2019-2020 would be another pandemic year (Yan et al., 2016). In the present study, we conducted a retrospective epidemiologic analysis of *M. pneumoniae* prevalence during the last five years to explore the characteristics of children with *M. pneumoniae* in Beijing (China), and to investigate the effect of the coronavirus disease 2019 (COVID-19) pandemic on the *M. pneumoniae* prevalence.

## Materials And Methods

### Patients and Clinical Specimens

A total of 569,887 pediatric patients with respiratory infection, who encountered at the outpatient/emergency department of Children’s Hospital Affiliated to Capital Institute of Pediatrics, Beijing, China between June 1, 2016 and May 31, 2021, were included in this study. The majority of cases were clinically diagnosed with upper respiratory infection, pneumoniae, bronchitis only, or respiratory infections associated with bronchiolitis, pleural effusion, atelectasis, lung abscess, central nervous system disorders. As a tertiary first-class general children’s hospital, over 90% outpatient/emergency patients with acute upper respiratory symptoms come from different regions of Beijing. The overall composition of patients can basically represent the whole Beijing area. These patients were aged 3.48 ± 2.65 (range, 0-17) years old, and they were divided into four age groups as follows: infants (<1 year old), toddlers (1-2 years old), preschoolers (3-6 years old), and school-age children (≥7 years old) (Yamazaki and Kenri, 2016). Peripheral blood was collected for routine blood test at the time of outpatient visit. It was a retrospective study, and all the patients’ data were anonymously reported in this study. It has been approved by the Ethics Committee of Capital Institute of Pediatrics.

### Detection

*M. pneumoniae* specific-IgM antibody was tested by a rapid immunochromatographic assay kit (Zhuhai Lizhu Reagent Co., Ltd., Zhuhai, China) with colloidal gold labeled anti-*M. Pneumoniae* monoclonal antibody for each serum specimen collected from the 569,887 patients according to the manual (Yan et al., 2019). Serum samples were added to the test strips with antigen. If there was a test line and a quality control line 5-10 minutes after the test, the result was considered positive. This test method was not significantly different from passive particle agglutination method with antibody titer ≥1:160. *M. pneumoniae* positive was defined as follows: along with symptoms of acute respiratory infection, i.e., fever and/or cough, *M. pneumoniae* specific-IgM antibody test was positive once, and repeated tests within three months only recorded once.

### Statistical Analysis

The positive rates of *M. pneumoniae* in diverse years, months, and patients of different genders and age groups were analyzed using RStudio software (version 1.4.1106). The Chi-square test was used to compare the differences between the two groups, and *p*<0.05 was considered statistically significant.

## Results

### Epidemiological Characteristics of *M. Pneumoniae* Over Years

**Figure 1:**
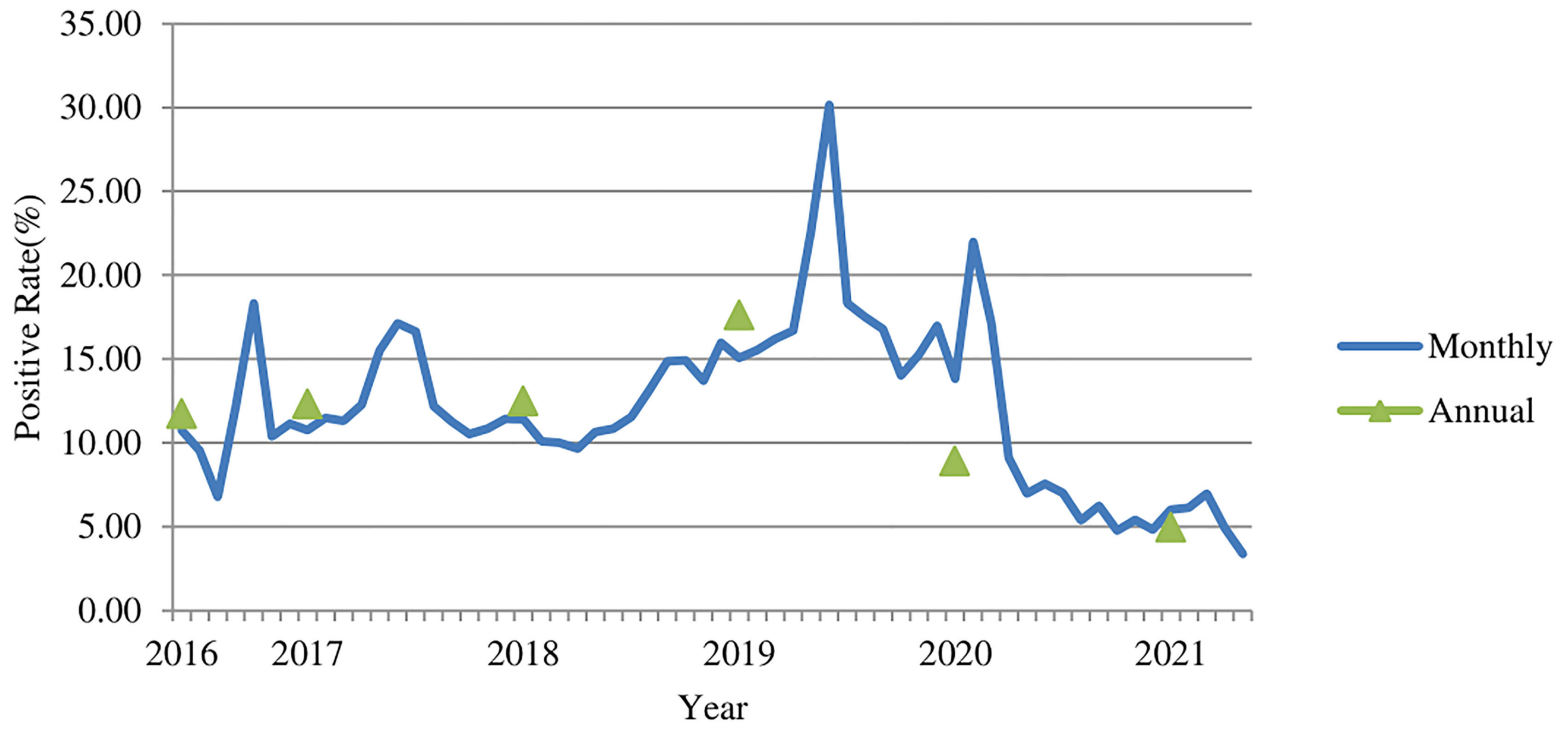
The *M. pneumoniae* IgM antibody positive rates (%) among children in Beijing from 2016 to 2021.

Of the 569,887 serum samples, 74,513 were positive for *M. pneumoniae* specific-IgM antibody. The average positive rate in the six years was 13.08%. The highest annual positive rate was 17.59% (26,350/149,795) in 2019, followed by 12.48% (18,286/146,519) in 2018, 12.31% (19,505/158,470) in 2017, and 11.73% (4,846/41,296) in 2016. The number of patients got tested for *M. pneumoniae* specific-IgM antibody in 2016 was low because the test just started to be used in outpatient department in 2016. The positive rate dropped to 8.9% (4,219/47,418) in 2020 (during the COVID-19 pandemic), and the lowest positive rate was 4.95% (1,307/26,389) in 2021 (**Figure 1**) , with significant difference (χ2 = 5140.2, *p*<0.001). The highest monthly positive rate in the six years was 30.17% (3,038/10,071) in June 2019, while the lowest positive rate was 3.37% (289/8,575) in May 2021 (χ2 = 2266.7, P<0.001).

### Epidemiological Characteristics of *M. Pneumoniae* in Different Seasons

The average positive rates in spring, summer, autumn, and winter seasons from 2016 to 2021 were 12.52% (15,088/120,503), 15.45% (15,250/98,727), 12.45% (20,176/162,121), and 12.73% (23,999/188,536), respectively. The average positive rates in summer and winter seasons were significantly higher than those in spring and autumn seasons (χ2 = 29.38, *p*<0.001), and the positive rate in the summer of 2019 was higher than that in other years (χ2 = 1410.2, *p*<0.001, **Table 1**).

**Table 1:** Positive rates (%) of *M. pneumoniae* specific-IgM antibody in serum samples stratified by season.

Year	Spring (Mar-May)	Summer (Jun-Aug)	Autumn (Sep-Nov)	Winter (Dec-Feb)	X^2^	*p*-value
**2016**	–	8.98	13.01	11.08	20.00	<0.001
**2017**	13.07	15.28	10.87	11.19	29.57	<0.001
**2018**	10.11	11.82	14.40	15.52	24.38	<0.001
**2019**	18.46	22.46	15.28	15.98	1231.7	<0.001
**2020**	11.93	6.23	5.37	5.38	201.79	<0.001
**2021**	4.57	–	–	–	–	–
**Average**	12.52	15.45	12.45	12.73	–	–
**X^2^**	2455.9	1784.5	1281.3	1632.7	–	–
***p*-value**	<0.001	<0.001	<0.001	<0.001	–	–

### Epidemiological Characteristics of *M. Pneumoniae* in Cases of Different Age Groups

**Figure 2:**
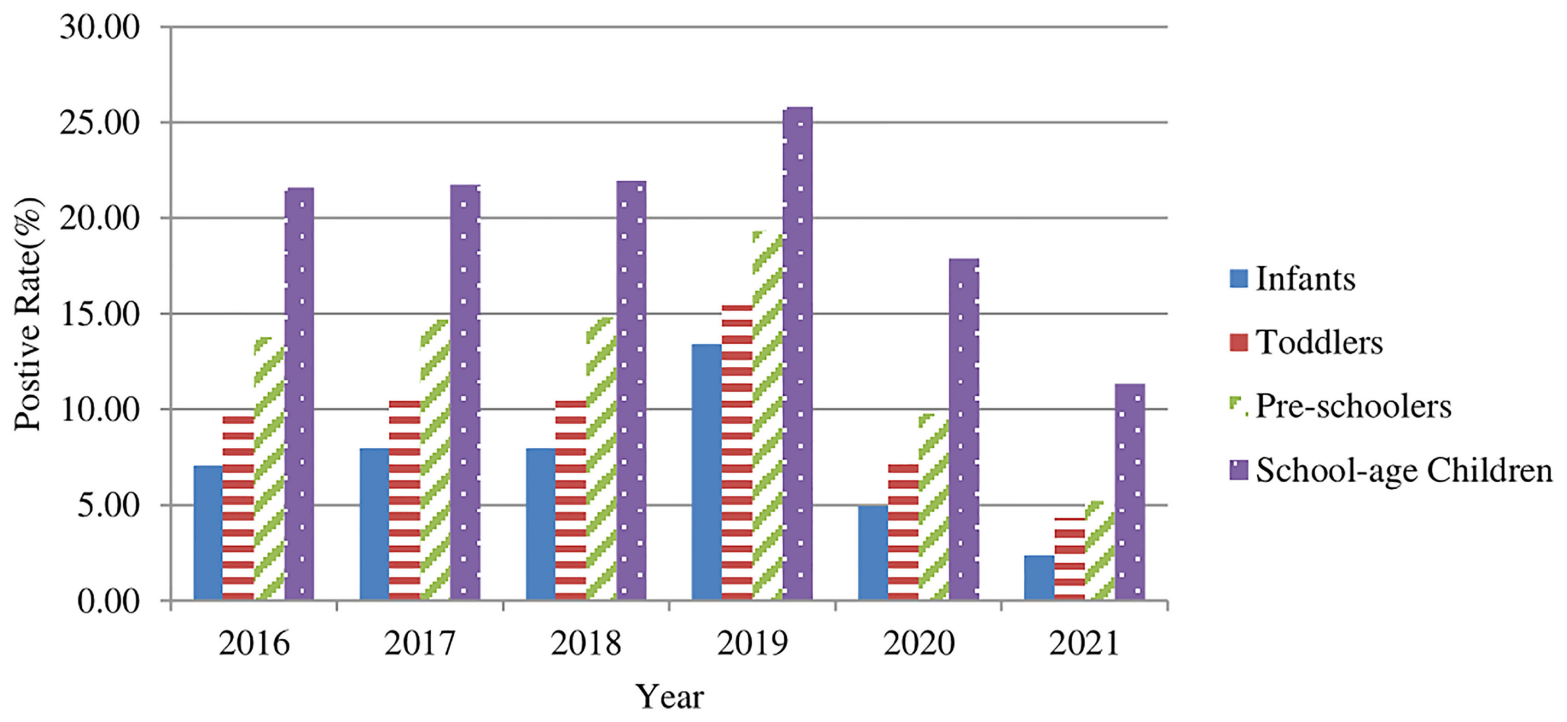
The *M. pneumoniae* IgM antibody positive rates (%) for children of different age groups from 2016–2021.

The 74,513 positive cases were aged 4.27 ± 2.81 (range, 0-17) years old. These cases were categorized into four groups as follows: infants (age<1 year old), toddlers (1-2 years old), preschoolers (3-6 years old), and school-age children (>=7 years old). The positive rates (%) of *M. pneumoniae* specific-IgM antibody in serum samples in the four groups were 8.76% (12,257/139,998), 11.31% (21,807/192,880), 15.02% (25,451/169,446), and 22.20% (14,998/67,563), respectively (**Figure 2**). The positive rates increased with the elevation of age, and the differences among the age groups were statistically significant (χ2 = 8342, *p*<0.001). However, there was no significant difference before and during the COVID-19 pandemic (*p*>0.05).

### Epidemiological Characteristics of *M. Pneumoniae* in Cases of Different Genders

Among the 74,513 positive cases, 36,929 (49.56%) cases were boys, and 37,584 (50.44%) were girls, with a gender ratio of 1:1. The positive rate (%) of *M. pneumoniae* specific-IgM antibody in serum samples in boys was 11.69% (36,929/315, 974), which was lower than that in girls (14.80%, 37,584/253, 913), with significant difference (χ2 = 1201.3, *p*<0.001). From 2016 to 2021, the annual positive rates in girls were all higher (13.01%, 13.87%, 14.17%, 19.81%, 10.38%, and 6.13%, respectively) than those in boys (10.71%, 11.06%, 11.14%, 15.78%, 7.70%, and 3.99%, respectively), with significant difference (*p*<0.001). There was also no significant difference before and during the COVID-19 pandemic (*p*>0.05).

## Discussion

*M. pneumoniae* is a common pathogen of respiratory infection, and it is responsible for 10-30% of community-acquired pneumoniae (CAP). In addition to the respiratory infection, it may also cause complications of nervous system, blood system, cardiovascular system, digestive system, etc. (Yan et al., 2016) The co-infection rate of Mycoplasma pneumoniae with other pathogens, especially with virus was high (Chiu et al., 2015; Kim et al., 2018; Wei et al., 2022), and the overall co-infection rate was 51.2% (Chen et al., 2012), so the co-infection patients were not excluded from our study. *M. pneumoniae* infection may occur in different seasons, and the positive rate may increase in summer, autumn, and winter seasons (Waites et al., 2017; Kuo et al., 2021). *M. pneumoniae* outbreaks occur every 3-7 years, and an outbreak may last for 1-2 years. The last two outbreaks of *M. pneumoniae* were in 2013 and 2016 (Lenglet et al., 2012; Yan et al., 2016). According to the pattern of *M. pneumoniae* epidemic, it was suggested that there might be an outbreak at three or more years after 2016. In our study, an *M. pneumoniae* outbreak was found in Beijing starting from summer 2019, with the highest annual positive rate (17.59%) of *M. pneumoniae* IgM after 2016 and the highest positive rate (22.46%) in the summer of 2019, especially the highest monthly positive rate (30.17%) was found in June 2019. According to the previous pattern of *M. pneumoniae* epidemic, this epidemic may last from summer and autumn of 2019 until winter of 2020 or spring of 2021. Nevertheless, the positive rates of *M. pneumoniae* IgM antibody in 2020 and 2021 were markedly lower than those in previous years, and our results showed that there was a substantial decrease in the positive rate since February 2020, which was kept at a relatively low level afterwards, with some fluctuations. The number of patients got tested in 2020 and 2021 was noticeably decreased because fewer patients came to visit clinicians after the COVID-19 pandemic outbreak and the restrictive measures responsive to COVID-19 reduced the incidence of infectious diseases of the respiratory tract. It was also reported that the number of positive cases of *M. pneumoniae* in Chengdu (China) decreased in the summer of 2020 (Kuo et al., 2021; Zhang Y. et al., 2021). Studies in Japan and Finland also demonstrated that the prevalence of *M. pneumoniae* was noticeably reduced in 2020 compared with 2012 and 2016 (Fujita, 2021; Haapanen et al., 2021). In the end of January 2020, right after the outbreak of COVID-19 in Wuhan, China, The National Health Commission issued six public prevention guidelines, including general, tourism, family, public places, public transportation, and home observation, to provide detailed measures to the public during the pandemic. These restrictive measures included wearing masks in public, sanitizing hands regularly, taking classes online, limiting the public parties, health monitoring, travel restrictions and border closures, etc. And it was the winter holiday for schools, so children had no close contact with other children. In March 2020, the beginning of the new spring semester, the Ministry of Education released the “Guidelines for the Prevention and Control of Novel Coronavirus Pneumoniae in Primary and Secondary Schools” to guide and assist the prevention and control of the epidemic in schools. All the measures above may have helped control the spread of respiratory pathogens in an effective manner, such as *M. pneumoniae* in 2020. This is consistent with the findings of Yeoh’s and Brueggemann’s studies that the COVID-19 restrictive measures had reduced the transmission of *M. pneumoniae* effectively (Brueggemann et al., 2021; Yeoh et al., 2021).

Our results revealed that the positive rate of *M. pneumoniae* in preschool and school-age groups was higher than that in the infant and toddler groups, which was consistent with previous studies (Zhang et al., 2015; Guo et al., 2019; Yan et al., 2019; Zhang L. et al., 2021). Besides, there were no significant differences before and during the COVID-19 pandemic (*p*>0.05). The school-age and preschool children would spend more time to study or play with other children in their same age groups in school or daycare settings, even in the community during COVID-19 pandemic, thereby facilitating *M. pneumoniae* transmission through spreading of droplets *via* close contacts in the same age group, which are consistent with previously reported findings. During the COVID-19 pandemic, the positive rates of all age groups were significantly reduced. The effective control measures of the COVID-19 pandemic could greatly reduce the prevalence of *M. pneumoniae* in semi-closed or closed communities, such as institutions, schools, religious communities, hospitals, and military bases (Suzuki et al., 2019; Zhang et al., 2019; Zhang et al., 2020).

The seasonal trend of *M. pneumoniae* infection annually varies (Chen et al., 2019; Yan et al., 2019; Lee et al., 2020). Some research indicated that higher positive rates were observed in autumn (August, September, and October) and winter seasons compared with spring and summer seasons (Zhao et al., 2014; Yan et al., 2019; Lee et al., 2020). In other studies, the prevalence of *M. pneumoniae* infection was higher in the summer season (Waites et al., 2017; Chen et al., 2019; Gayam et al., 2020), and the prevalence of *M. pneumoniae* infection was positively correlated with the increase of temperature (Onozuka et al., 2009; Waites et al., 2017). In our study, the highest monthly positive rate (30.17%) was found in June 2019, and the positive rates in summer and winter (13.66%) seasons were higher than those in spring and autumn seasons (12.48%, *p*<0.001). The seasonal variation of *M. pneumoniae* infection may be related to global warming and some other climate factors, such as temperature, humidity, El Nino events, etc. (Onozuka et al., 2009). People prefer to gather in closed or semi-closed air-conditioned environments in summer, which might assist the transmission of M. pneumoniae. The extremely cold and hot weather may also have a certain influence on individuals’ immune functions. On the other hand, it was suggested that there might be an *M. pneumoniae* epidemic in 2019 (Yan et al., 2016), and there might be two peaks during *M. pneumoniae* epidemic, one in autumn/winter and one in summer (Yan et al., 2019). The high prevalence in summer 2019 might indicate a new epidemic of *M. pneumoniae*, but due to the restrictive measures responsive to the COVID-19 pandemic, we did not see a high prevalence in winter 2019/2020. But long-term observation is required to better investigate the seasonal trend of *M. pneumoniae* infection after COVID-19.

In our study, the difference in gender-based variations was noticeable, and girls were more susceptible to *M. pneumonia*e than boys, which was consistent with the findings of some previous studies (Qian et al., 2010; Zhang et al., 2015; Yan et al., 2019). This may be related to different lifestyles of girls and boys. Boys may spend more time doing outdoor activities than girls.

In conclusion, this study demonstrated that an *M. pneumoniae* outbreak started from the summer of 2019 in Beijing after the one occurred in 2016. After the outbreak of COVID-19 pandemic at the end of 2019, the positive rates of *M. pneumoniae* dropped dramatically. This may be due to the restrictive measures of the COVID-19 pandemic, which successfully controlled *M. pneumoniae* transmission. The positive rate of *M. pneumoniae* in summer and winter seasons was relatively higher compared to spring and autumn seasons. Thus, an appropriate non-drug preventive isolation, i.e. wearing masks, sanitizing hands regularly, avoiding going to crowded places, is recommended in the years of *M. pneumoniae* epidemic, especially in the epidemic seasons, in order to control the *M. pneumoniae* outbreak, even after the COVID-19 pandemic. There were no significant differences before and during the COVID-19 pandemic regarding the susceptibility of girls and older children to *M. pneumoniae*.

There were some limitations in our study. First, the positive rate was investigated by detecting the *M. pneumoniae* specific-IgM antibody, and no nucleic acid test was performed. As the IgM antibody test results may be influenced by the time and duration of antibody production, the nucleic acid test combined with antibody detection is more reliable for the diagnosis (Expert Committee on Rational Use of Medicines for Children Pharmaceutical Group and National Health and Family Planning Commission, 2020). Secondly, a longer follow-up after the COVID-19 pandemic is needed to better understand the epidemiology of *M. pneumoniae* during and after the COVID-19 pandemic.

## Data Availability Statement

The raw data supporting the conclusions of this article will be made available by the authors, without undue reservation.

## Ethics Statement

This study has been approved by the Ethics Committee of Capital Institute of Pediatrics. It was a retrospective study, and all the patients’ data were anonymously reported in this study. Based on the guidelines of the Ethics Committee of the Capital Institute of Pediatrics, informed consent was not sought from patients.

## Author Contributions

YC, GX, LM, and HS contributed to conception and design of the study. YJC performed the statistical analysis and drafted the manuscript. SD, DH, MG, YZ, LF, YP, and LY read and revised the manuscript. All authors contributed to the article and approved the submitted version.

## Conflict of Interest

The authors declare that the research was conducted in the absence of any commercial or financial relationships that could be construed as a potential conflict of interest.

## Publisher’s Note

All claims expressed in this article are solely those of the authors and do not necessarily represent those of their affiliated organizations, or those of the publisher, the editors and the reviewers. Any product that may be evaluated in this article, or claim that may be made by its manufacturer, is not guaranteed or endorsed by the publisher.
